# Identification and validation of ferroptosis-related hub genes in obstructive sleep apnea syndrome

**DOI:** 10.3389/fneur.2023.1130378

**Published:** 2023-03-02

**Authors:** Peijun Liu, Dong Zhao, Zhou Pan, Weihua Tang, Hao Chen, Ke Hu

**Affiliations:** ^1^Department of Respiratory and Critical Care Medicine, Renmin Hospital of Wuhan University, Wuhan, China; ^2^Department of Radiology, The Central Hospital of Enshi Tujia and Miao Autonomous Prefecture, Enshi, China

**Keywords:** OSAS, THCA, ferroptosis, CIH, HIF1A, ATM, immune infiltration

## Abstract

**Background:**

By 2020, the prevalence of Obstructive Sleep Apnea Syndrome (OSAS) in the US has reached 26. 6–43.2% in men and 8.7–27.8% in women. OSAS promotes hypertension, diabetes, and tumor growth through unknown means. Chronic intermittent hypoxia (CIH), sleep fragmentation, and increased pleural pressure are central mechanisms of OSAS complications. CIH exacerbates ferroptosis, which is closely related to malignancies. The mechanism of ferroptosis in OSAS disease progression remains unknown.

**Methods:**

OSAS-related datasets (GSE135917 and GSE38792) were obtained from the GEO. Differentially expressed genes (DEGs) were screened using the R software and intersected with the ferroptosis database (FerrDb V2) to get ferroptosis-related DEGs (f-DEGs). GO, DO, KEGG, and GSEA enrichment were performed, a PPI network was constructed and hub genes were screened. The TCGA database was used to obtain the thyroid cancer (THCA) gene expression profile, and hub genes were analyzed for differential and survival analysis. The mechanism was investigated using GSEA and immune infiltration. The hub genes were validated with RT-qPCR, IHC, and other datasets. Sprague-Dawley rats were randomly separated into normoxia and CIH groups. ROS, MDA, and GSH methods were used to detect CIH-induced ferroptosis and oxidative stress.

**Results:**

GSEA revealed a statistically significant difference in ferroptosis in OSAS (FDR < 0.05). HIF1A, ATM, HSPA5, MAPK8, MAPK14, TLR4, and CREB1 were identified as hub genes among 3,144 DEGs and 74 f-DEGs. HIF1A and ATM were the only two validated genes. F-DEGs were mainly enriched in THCA. HIF1A overexpression in THCA promotes its development. HIF1A is associated with CD8 T cells and macrophages, which may affect the immunological milieu. The result found CIH increased ROS and MDA while lowering GSH indicating that it could cause ferroptosis. In OSAS patients, non-invasive ventilation did not affect HIF1A and ATM expression. Carvedilol, hydralazine, and caffeine may be important in the treatment of OSAS since they suppress HIF1A and ATM.

**Conclusions:**

Our findings revealed that the genes HIF1A and ATM are highly expressed in OSAS, and can serve as biomarkers and targets for OSAS.

## Introduction

Obstructive sleep apnea syndrome (OSAS) is one of the most frequent chronic respiratory disorders, affecting almost a billion people globally and having devastating effects on both individuals and society (1). OSAS can increase the prevalence of neurological disorders such as malignant tumors, coronary heart disease, pulmonary heart disease, diabetes, vestibular abnormalities, and depression (2–4). OSAS is characterized by recurrent full or incomplete pharyngeal collapse during sleep, leading to chronic intermittent hypoxia (CIH) and sleep fragment, with a recent trend toward younger onset, particularly in infants with congenital developmental abnormalities (5, 6). OSAS-induced intermittent hypoxia and sleep fragment increase cancer or its aggressiveness, as well as the occurrence of antitumor therapy resistance (7). Furthermore, OSAS-related repeated upper airway obstruction can cause physical discomfort due to prolonged CIH (8, 9). OSAS is an individually variable condition with diverse symptoms and endotypes, the focus of this study is the pathophysiology and mechanisms of sleep breathing problems (10).

Ferroptosis is a new kind of iron-dependent cell death described by Dixon in 2012 that is morphologically distinct from apoptosis and autophagy (11). The morphological features of ferroptosis are mainly damaged cells with intact cell membranes, increased mitochondrial membrane density, reduced or absent mitochondrial cristae, mitochondrial membrane shrinkage, and outer membrane rupture. The chromatin of cells is not condensed, and their nuclei are of normal size (12). The genes associated with ferroptosis can be categorized based on six modules: Drivers, Suppressors, Markers, Inducers, Inhibitors, and Diseases (13).

Ferroptosis is essential for the occurrence and development of pathological processes and diseases that include cerebral hemorrhage, ischemic stroke, sepsis, cancer, and myocardial infarction (14). Ferroptosis has demonstrated tremendous promise as a cancer therapy, and OSAS not only relates to metabolic and cardiovascular illnesses but also to the progression of cancer (15). OSAS is a prevalent form of respiratory illness (16). There are few findings on the association between ferroptosis and OSAS, but there are numerous correlation studies between ferroptosis and other respiratory illnesses. Several recent studies have shown that ferroptosis is a potential therapeutic target for lung diseases including acute lung injury, chronic obstructive pulmonary disease (COPD), pulmonary fibrosis (PF), lung infection, and asthma (17). Numerous animal and cellular models of acute lung injury (ALI) have established the role of ferroptosis in the course of the disease (18).

Through the promotion of autophagy, reactive oxygen species (ROS) are able to upregulate both ferroptosis and intracellular ferritin (19). CIH can cause an increase in ROS, which can lead to cellular ferroptosis as a possible consequence (20). ROS/HIF1A leads to increased oxidative stress and increases systemic inflammation, whereas inflammation can also enhance HIF1A expression and aggravate the oxidative stress reaction; these two phenomena are closely connected (21). HIF1A is both a regulatory protein and a transcription factor in the molecular physiology of oxygen homeostasis (19). HIF1A regulates multiple glycolysis, proliferation, invasion, and survival genes in response to hypoxia (22, 23). There is a significant correlation between HIF1A and lung cancer, and the expression of HIF1A in non-small cell lung cancer is mediated by the AKT and ERK signaling pathways (24).

Because the lungs are in a hyperoxic physiological state when compared to other human organs, the pathophysiology of obstructive sleep apnea is mostly manifested as CIH, which is unique from other lung diseases that are in a permanent hypoxic state (25). Oxidative stress has the potential to produce a significant number of ROS, the accumulation of which is one of the primary processes that contribute to the promotion of ferroptosis. This may be very useful for understanding the function of ferroptosis in OSAS (26). As a consequence of this, we have developed the research hypothesis that ferroptosis plays an important role in OSAS. This prompted us to perform bioinformatics and CIH animal experiments to investigate the relationship between OSAS, tumors, and ferroptosis.

## Materials and methods

### Acquisition of datasets and RNA degradation

The RNAseq data (GSE135917 and GSE38792) related to OSAS were downloaded from the GEO database (https://www.ncbi.nlm.nih.gov/geo/) (27, 28). Both of their sequencing platforms are GPL6244, total RNA was isolated from human subcutaneous fat. The first group of GSE135917 consisted of 10 OSAS patients and 8 normal people, whereas the second group consisted of 24 OSAS patients who were sampled individually after treatment with a continuous positive airway pressure (CPAP) ventilator. In brief, CPAP was started following the biopsy and after 2 weeks of self-reported CPAP use for more than 4 h per night. The follow-up biopsy was conducted on the other side of the abdomen.

The ReadAffy function from the affy package (version 1.72.0) was utilized to read the raw data included in the cell format files. The data were imported into SIMCA 14.1 (Sartorius, Malmö, Sweden) for analysis, and four components were chosen to construct the PLS-DA model (29, 30).

### F-DEGs, GSEA, and GSVA

The raw data are normalized using the Affymetrix platform in R software (version 4.0.5). The RMA functions were then used to process data using the affy package. The Limma package (version 3.50.3) was used to perform DEGs between OSAS and normal groups with the empirical bayesian *t*-test, and |log2-Fold change (FC)| >0.5 and *P* < 0.05 were utilized as DEG screening criteria. The heatmap package was used to create a heat map that displayed the top 50 genes with the most significant genes. The ferroptosis-related genes were downloaded from the FerrDb V2 database (http://www.zhounan.org/ferrdb/), which mainly contained gene sets such as “Driver,” “Suppressor,” and “Marker.” A Venn diagram was utilized to illustrate the intersection genes of FerrDb V2 and DEGs (f-DEGs) (31).

The genes in the dataset were ranked by OSAS phenotype and logFC value, and Gene Set Enrichment Analysis (GSEA) was performed on 664 gene sets from WikiPathways. The statistical differences were determined by the normalized enrichment score (|NES| >1), and FDR < 0.25. The dataset was read and subjected to Gene set variation analysis(GSVA) analysis using the GSVA package (version 1.42.0) to acquire a GSVA score for each sample. The Limma package was used to compare the GSVA scores between the OSAS and the normal group with the Bayesian *t*-test, adj. *P* < 0.05 and |logFC| > 0.1 represent significant differences.

### Screening F-DEGs biomarkers

The f-DEGs were transformed with the org.Hs.eg.db package (version 3.10.0) and then enriched with the cluster profiler package (version 4.2.2) for gene ontology, disease ontology, and Kyoto Encyclopedia of Genes and Genomes (KEGG) pathway analysis (32, 33). The q value ≤ 0.05 and gene counts ≥3 were considered significant.

### Construction of PPI network and identification of hub genes

The f-DEGs were analyzed using the STRING database (https://cn.string-db.org/) and a combined interaction score>0.4 was considered statistically significant. Subsequently, the string interactions were visualized using Cytoscape software (version 3.9.0). CytoNCA (2.1.6), a Cytoscape plug-in, was used to filter the density and importance of modules in the PPI networks. Then, the R software screened out the hub genes in the network, based on the following principles: betweenness, closeness, and degree are greater than average.

### The hub gene of F-DEGs analysis in THCA

The THCA gene expression profiles were downloaded from the TCGA database. There were 568 cases of TCGA (510 cases in the tumor group and 58 cases in the normal group). The online database ULCAN (http://ualcan.path.uab.edu) analyzed the gene expression level of HIF1A in cancer tissues and normal tissues of TCGA database samples. Validation of the effect of HIF1A expression on THCA survival through the Kaplan-Meier plotter (http://kmplot.com/analysis/). Transcriptome data for THCA were grouped according to pathology type or gene expression. CIBERSORT was used to compute immune cell composition based on gene expression profiles. R software (including vioplot, ggpubr, and ggExtra packages) was used to analyze the correlation between HIF1A and THCA immune infiltration.

### Immune infiltration in OSAS

In the GSE135917 dataset, single sample gene set enrichment analysis was used to calculate the per sample infiltration levels of immune cell types. The ggboxplot function in the ggpubr package (version 0.4.0) was used to plot box line plots of immune cells in the two groups of samples. The heatmaps were plotted using the pheatmap package (version 1.0.12), showing the relationship between hub genes and various immune cells.

### F-DEGs validation in GSE38792 and animal model

GSE38792 was utilized to validate the differential expression of hub genes between OSAS patients and healthy volunteers. A variance analysis was performed using the stat_compare_means function in the ggpubr package (version 0.4.0), and the results were presented in box plots (34).

Eight male Sprague-Dawley rats (SPF grade, weight of 230–250 g) were obtained from the central laboratory of the Animal Experimental Center at Renmin Hospital of Wuhan University. The rats were fed in a 12-h alternating day and night environment with corresponding humidity and temperature control. The rats were randomly divided into two groups: (1) Normal control group, in which the rats were kept in a normoxic environment for 8 weeks; (2) Chronic intermittent hypoxia group (CIH), in which the mice were kept in a chronic intermittent hypoxic environment 8 h per day for 8 weeks. The oxygen concentration in the modeling chamber alternated between 30 s of hypoxia (FiO2, 10%) and 60 s of reoxygenation (FiO2, 21%). After 8 weeks, we administered general anesthesia to rats, isolated the abdominal aorta, and obtained arterial blood for arterial blood gas analysis using a blood gas needle.

### Reverse transcription and real-time PCR analysis

Total RNA was isolated and purified using RNAiso Plus reagent (9108, Takara, Japan) according to the manufacturer's protocol. Based on the TaqMan probe method, The mRNA expressions of hub genes (HIF1A, ATM, MAPK8, and MAPK14) were detected according to the instructions of probe One-Step qRT-PCR Kit (D7277, Beyotime, China). The reactions were carried out in the cycler under the following conditions: 50°C for 20 min, 95°C for 2 min, 95°C for 15 s and 60°C for 20 s (40 cycles in total). GAPDH was used as a housekeeping gene, and the RT-qPCR primer sequences are listed in Table 1. The relative gene expression level was calculated using the 2– ΔΔCT method (35).

**Table 1:** The primers for qRT-PCR.

**Target**	**Forward primer (5'-3')**	**Reverse primer (3'-5')**	**bp**
Hif1a (Rat)	AAGCAGCAGGAATTGGAACG	CTCGTTTCCAAGAAAGCGACA	75
Atm (Rat)	CAGCTTTAGAGAGGTGTGTAATGA	AAGTCTCTGCCAGCCAGTTG	89
Mapk8 (Rat)	ACAGCTCGGAACACCTTGTC	TCGCCTGACTGGCTTTAAGT	167
Mapk14 (Rat)	GCACTACAACCAGACAGTGGA	GTCCCCGTCAGACGCATTAT	129
GAPDH (Rat)	CCGCATCTTCTTGTGCAGTG	CGATACGGCCAAATCCGTTC	79

### ROS measurements and ELISA assay

ROS production was detected by *in situ* staining of fresh lung tissue with a ROS fluorescent probe-dihydroethidium (DHE, D7008, Sigma, 1:500). In this process, sections incubated with stain sections were observed under a fluorescence microscope (IX53, Olympus, Japan). Fluorescein-labeled ROS-positive sections emitted red fluorescence (excitation wavelength 490 nm, emission wavelength 560 nm). At least three sites were selected for each sample. The fluorescence intensity of the cell sections was measured using Image J software.

The lungs were quickly removed after the rats were anesthetized and sacrificed. The saline was rinsed and blotted dry with filter paper. The lung tissue was ground with a cryogenic grinder at 4°C, and the supernatant was centrifuged and assayed according to the manufacturer's instructions for the MDA and GSH kits (A001-3-1/A006-2-1, Nanjing Jiancheng Bioengineering Institute, China). The contents of MDA and GSH were measured in the normal and CIH groups for intra-assay and inter-assay repeated experiments, respectively. The intra- and inter-coefficients of variation were both < 10%.

### Immunohistochemistry

After the inguinal fat was removed from the rat, the adipose tissue was pressed with oil-absorbing paper (36). The expressions of HIF1A and Atm in rat inguinal adipose tissue were detected using immunohistochemistry. The sections (6 μm) were processed with deparaffinized and rehydration using xylene and different concentration gradients of ethanol. The sections were added to citric acid antigen repair solution (pH 6.0) and then heated in a 95°C water bath for 20 min for antigen repair (37). The primary antibodies used were HIF1A (1:500, K000487P, Solarbio, China), ATM (1:200, K009314P, Solarbio, China). The sections were incubated with HRP-labeled secondary antibodies for 1 h at room temperature. The relative IOD of the immune sections was measured with ImageJ software.

### ROC curve, hub genes relationships, and drug therapy OSAS prediction

We estimated the area under the ROC curve (AUC) using version 1.8 of the standard pROC tool for the R software. The Pearson correlation coefficient was used to conduct correlation analysis on the hub genes. The CPAP-treated population belongs to the second group in the dataset GSE135917. The paired *t*-test was performed to compare the variation in hub genes expression before and after the application of a non-invasive ventilator. The DrugBank database was used to search for probe targets (Version 4.2) (38).

### Statistics analysis

GraphPad Prism 8 statistical software (La Jolla, CA, USA) was utilized for statistical analyses. All results were expressed as a mean ± standard deviation (SD) from three independent experiments. The data were tested with the Shapiro-Wilk normality test and the variance homogeneity test. Comparisons were tested using paired or unpaired *t*-tests and the level of confidence was set at 95% (*P* < 0.05).

## Results

### The flowchart of the research and animal model of OSAS

**Figure 1:**
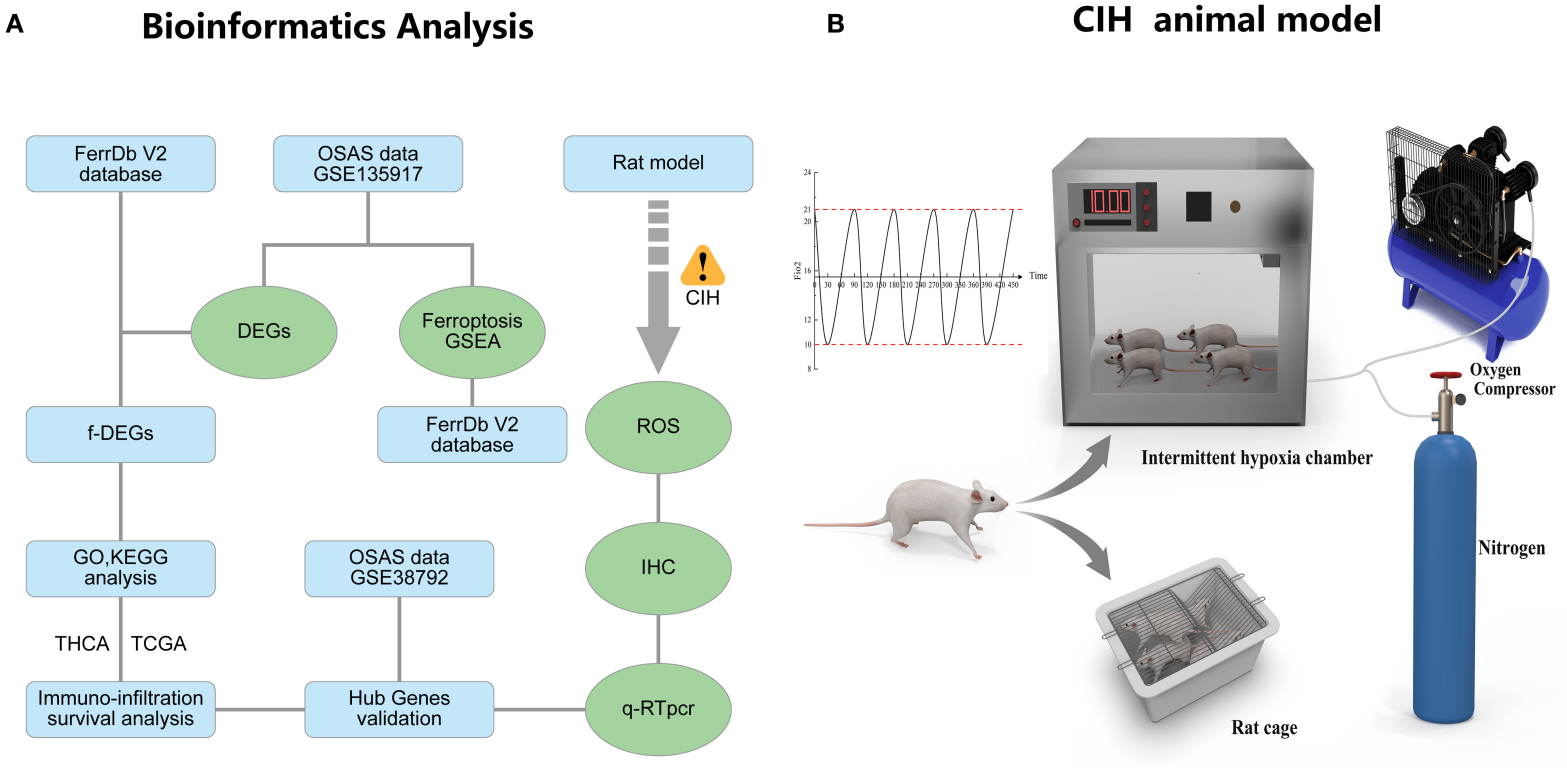
The research flowcharts and animal models. **(A)** Flowchart of bioinformatics analysis and experimental validation. **(B)** The experimental model of chronic intermittent hypoxia in rats.

Figure 1A depicts the whole study methodology, including bioinformatics analysis and rat validation model. The animal model of OSAS consists of three components: an intermittent hypoxic chamber, a nitrogen tank, and an oxygen compressor that varies FiO2 between 10 and 21% (Figure 1B).

### RNA degradation and PLS-DA model

To evaluate the accuracy of the gene sequencing in the GSE135917 dataset, we employed RNA degradation curve analysis and PLS-DA. The modest slope of each curve shows that mRNA is not degraded explicitly (Supplementary Figure S1A). The 2D and 3D structure of the PLS-DA model is depicted in Supplementary Figures S1B, C. Four components were used to construct the PLS-DA model, which had a cumulative explanatory power of 99.4% and a cumulative predictive power for the dependent variable of 73%. To determine if the PLS-DA model was overfitted, the model was evaluated by holding the X matrix constant and randomly rearranging the Y matrix variables 200 times to generate permutation test results (Supplementary Figure S1D). The results indicate that the normal and OSA groups can be discriminated against without difficulty and that the model is not overfitting.

### Identification of DEGs and GSEA, GSVA enrichment analysis

After GSE135917 Validation, we filtered the DEGs and performed GSEA and GSVA. A total of 23,281 gene expression values were gathered. The OSAS samples contained 1,470 down-regulated genes and 1,674 up-regulated genes, as compared to the normal controls.

**Figure 2:**
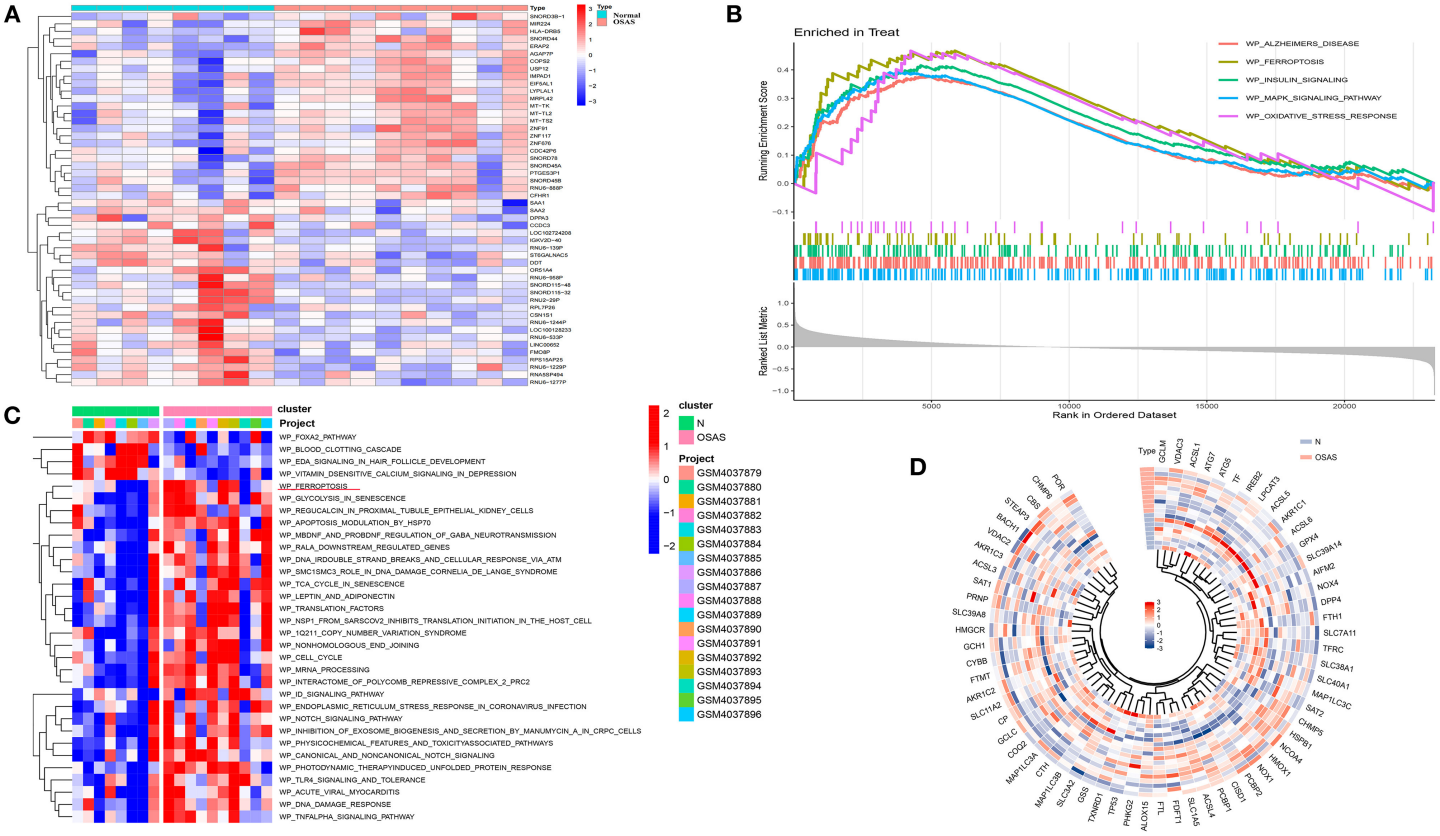
Identification of the DEGs in the GSE135917 dataset, GSEA analysis, and GSVA heatmap analysis. **(A)** Heatmap plot of the DEGs for OSAS vs. controls. **(B, C)** GSEA and GSVA analysis in wikipathways for OSAS vs. controls. (|NES| > 1, FDR < 0.1, *P-*value < 0.05). **(D)** Ferroptosis genes ring heatmap.

The heat map depicts the 50 genes with the greatest differences (positive and negative values) according to logFC values in the dataset (Figure 2A). Ferroptosis, Alzheimer's disease, insulin signaling, mapk signaling pathway, and oxidative stress response were all significantly different between the OSAS and normal groups. The ferroptosis pathway was significantly different between the two groups (NES = 1.8234, *P* = 0.0005, and FDR = 0.005) (Figure 2B). The WikiPathways were evaluated using gene set variation analysis, and it was determined that there was a significant difference in ferroptosis between the two groups, with elevated sample scores in the OSAS group (Figure 2C). The ferroptosis gene set included 64 genes, and the circular heat map revealed that GCLM, HMGCR, SLC38A1, and CHMP5 were elevated in OSAS (Figure 2D). This grants the theory that ferroptosis may play a significant role in the pathophysiology of OSAS.

### F-DEGs enrichment analysis

**Figure 3:**
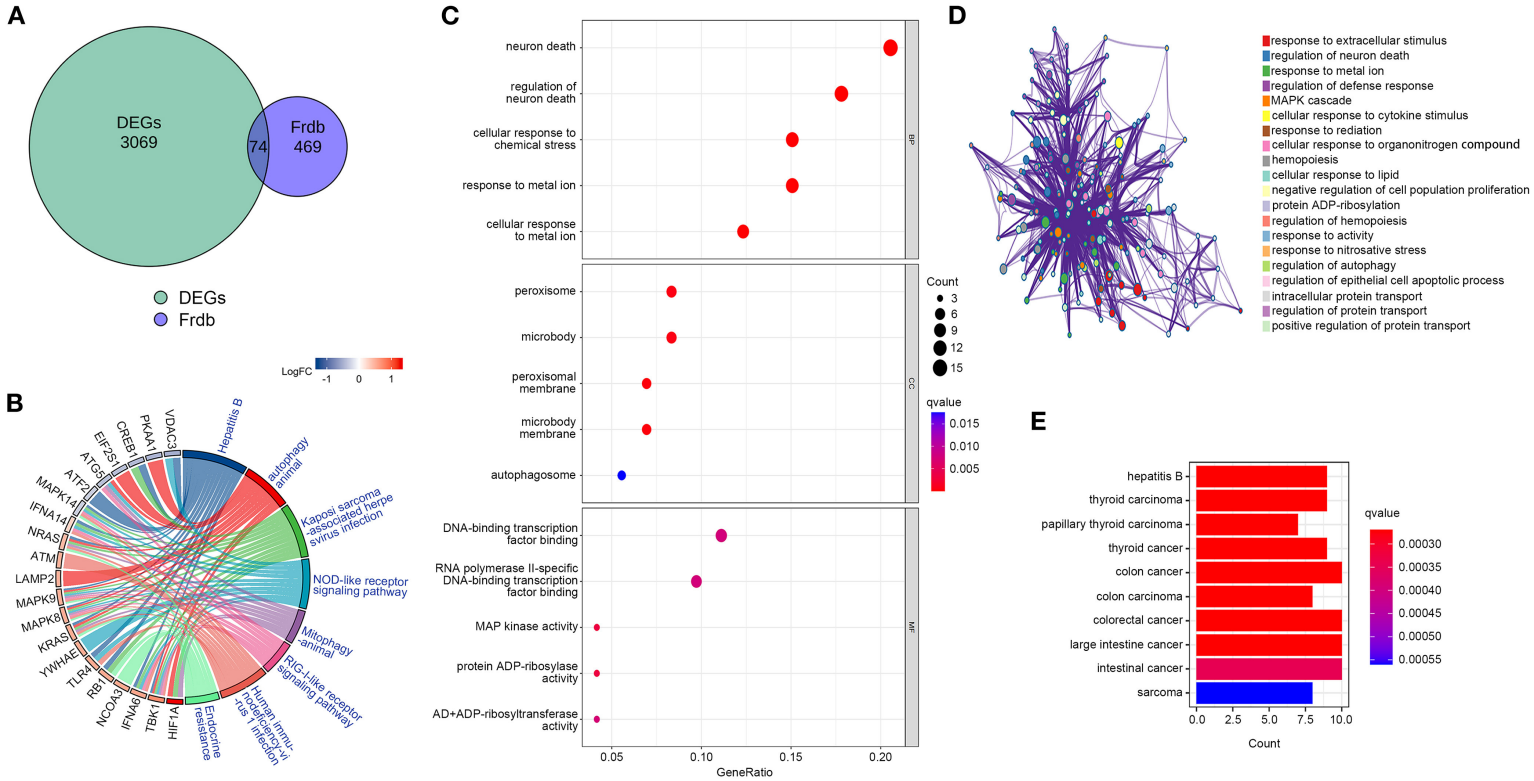
Identification of f-DEGs,f-DEGs enrichment analysis. **(A)** 74 f-DEGs were obtained from the intersection of the FerrDb V2 database with DEGs. **(B)** KEGG pathway analysis of f-DEGs. **(C)** GO enrichment analysis f-DEGs. **(D)** GO term analysis on f-DEGs using the Metascape website. **(E)** Disease Ontology analysis of f-DEGs.

To determine whether DEGs are involved in ferroptosis, we retrieved f-DEGs from the FerrDb V2 database and performed functional enrichment on them. The intersection of 543 ferroptosis-related genes from the FerrDb V2 database with DEGs using Venn diagrams generated 74 f-DEGs (Figure 3A). The KEGG pathway database was used to identify 102 enriched pathways, including ferroptosis, endocrine resistance, mitophagy-animal, autophagy-animal, and Kaposi's sarcoma-associated herpesvirus infection (Figure 3B, Supplementary Table S1).

The most enriched pathways in terms of GO terms were those associated with neuron death, regulation of neuron death, cellular response to metal ions, cellular response to chemical stress, and reaction to metallic ions (Figure 3C). On the Metascape website (https://metascape.org/), GO analysis of f-DEG revealed that ferroptosis-related pathways are involved in the regulation of neuronal death (Figure 3D). The f-DEGs were evaluated in the Disease Ontology database, and thyroid carcinoma, papillary thyroid carcinoma, thyroid cancer, and colon cancer were discovered to be enriched disorders (Figure 3E). We discovered that f-DEGs are closely associated with THCA and may play an important role in its onset.

### Identification of hub genes

**Figure 4:**
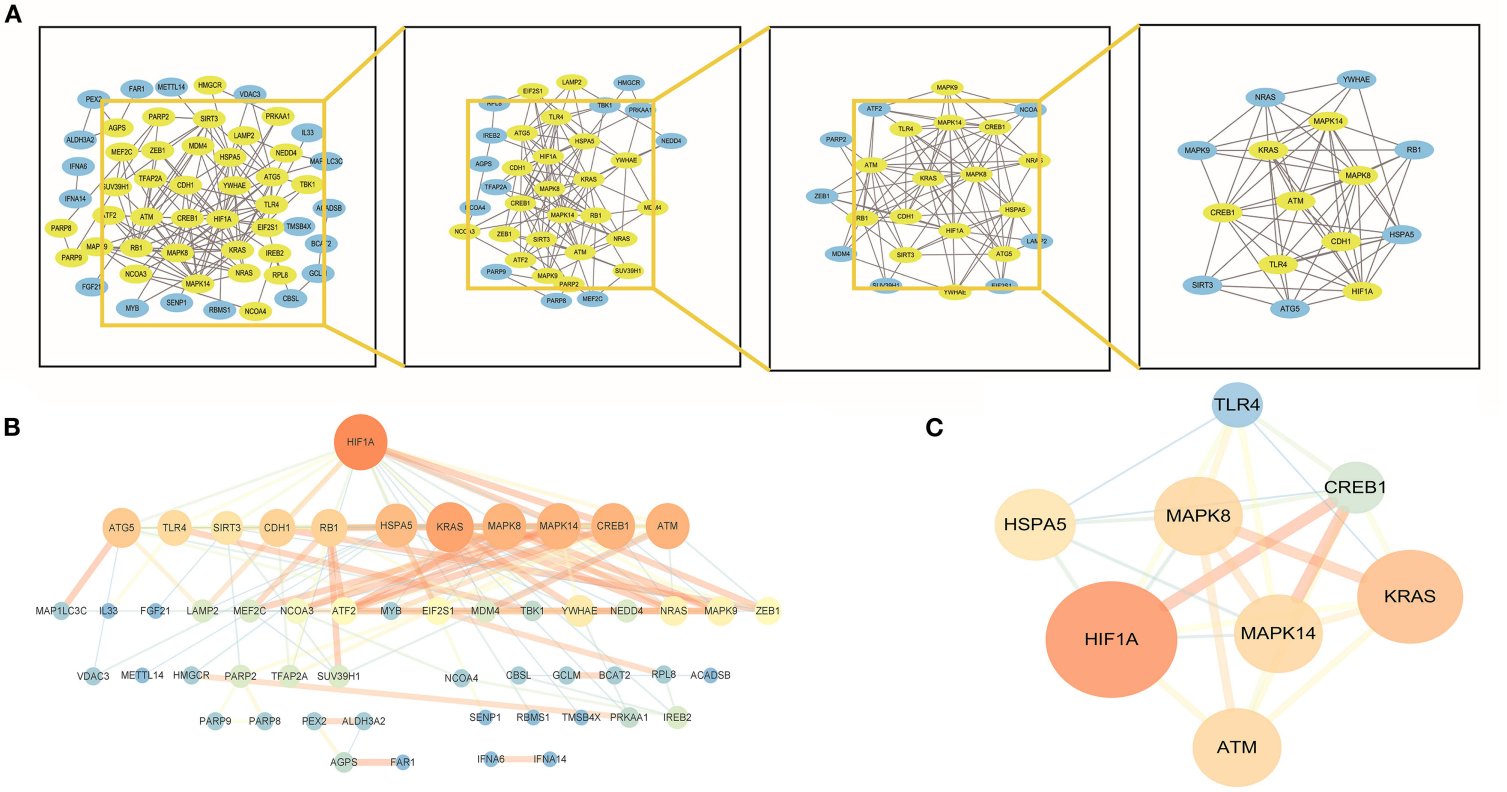
PPI network and cytoscape to obtain hub genes. **(A, B)** F-DEGs were analyzed with a PPI network and visualized with Cytoscape. **(C)** Hub network construction of HIF1A, ATM, MAPK14, TLR4, HSPA5, MAPK8, and CREB1.

By establishing a PPI network, we were able to identify the hub genes in f-DEGs and establish their relationship. A 71-node, 142-edge PPI network was constructed based on the biological interactions of 74 f-DEGs. The PPI enrichment P-value is 9.9910-16, and the average local clustering coefficient is 0.419. CytoNCA (2.1.6) was applied to analyze f-DEGs, resulting in a network of 53 nodes and 284 edges (Figures 4A, B). Furthermore, the hub genes were discovered using the same screening method: HIF1A, ATM, HSPA5, MAPK14, KRAS, MAPK8, TLR4, and CREB1 (Figure 4C). In the network, HIF1A is the most significant gene. Since f-DEGs are primarily enriched in THCA (Figure 3), we hypothesize that HIF1A may be the principal gene of THCA.

### HIF1A over expression promotes THCA progress

**Figure 5:**
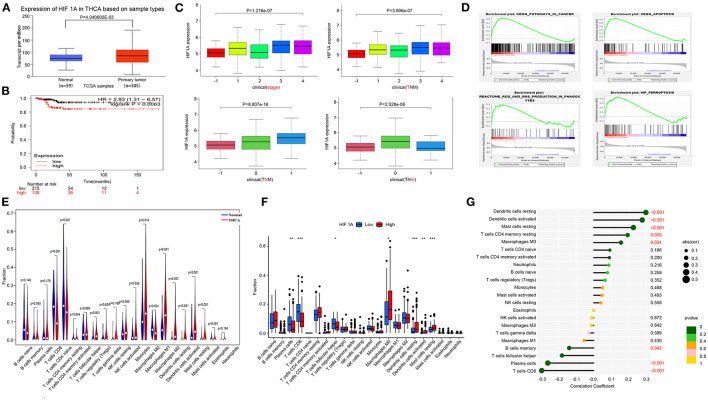
Increased expression of HIF1A promotes the progression of THCA. **(A)** The HIF1A expression difference in the tumor group vs. the normal group. **(B)** Kaplan-Meier plot illustrating the influence of HIF1A expression level on THCA survival. **(C)** HIF1A and TNM staging of thyroid cancer. **(D)** GSEA analysis of THCA pathway differences (FDR < 0.25 is deemed significant). **(E–G)** Correlation of THCA and HIF1A with immunological infiltration. *P < 0.05, **P < 0.01, ***P < 0.001.

We investigated the association between HIF1A and THCA, which was obtained from the TCGA database. The effect of high HIF1A expression in OSAS on thyroid cancer survival discovered that HIF1A expression in thyroid tissue was also increased, and patients with high HIF1A expression had a lower survival time. As a result, HIF1A overexpression in OSAS may accelerate the occurrence and progression of thyroid cancer (Figures 5A, B). This research examines the relationship between HIF1A, TNM, and thyroid cancer stage to determine the impact of HIF1A on thyroid cancer. HIF1A expression is lowest in normal people and increases with the T and N stages. In the M stage, the 0 stage was highest. HIF1A promotes thyroid carcinoma in patients (Figure 5C). To investigate whether HIF1A promotes THCA, the enrichment pathway was examined. HIF1A is enriched in the ferroptosis, ROS, cancer, and apoptosis pathways of THCA (Figure 5D). The immune milieu plays a crucial role in the development of tumors. HIF1A expression is intimately associated with dendritic cells, macrophages, mast cells, and CD8 T cells in the immunological milieu of THCA (Figures 5E, G). HIF1A causes alterations in the immunological microenvironment of thyroid cancer, which may be associated with ferroptosis of thyroid cells produced by an increase in ROS. OSAS potentially promote the onset and progression of THCA by increasing HIF1A expression.

### Immune infiltration in OSAS

We performed immune cell analysis on the GSE135917 dataset to understand the role of OSAS on the immune environment of adipocytes. OSAS was associated with an increase in activated CD4 T cells, gamma delta T cells, and regulatory T cells (Supplementary Figure S2A). A correlation heat map was generated based on the immune cell contact relationship (Supplementary Figure S2B). Analysis of the relationship between hub genes and immune cells demonstrated that HIF1A was the most important gene in OSAS immune cells (Supplementary Figures S2C, D). According to Figure 5 and Supplementary Figure S2, we found that HIF1A is critical for the development of THCA and OSAS, which may increase the risk of disease by affecting the immune microenvironment.

### Hub genes validation and CIH experimental validation

**Figure 6:**
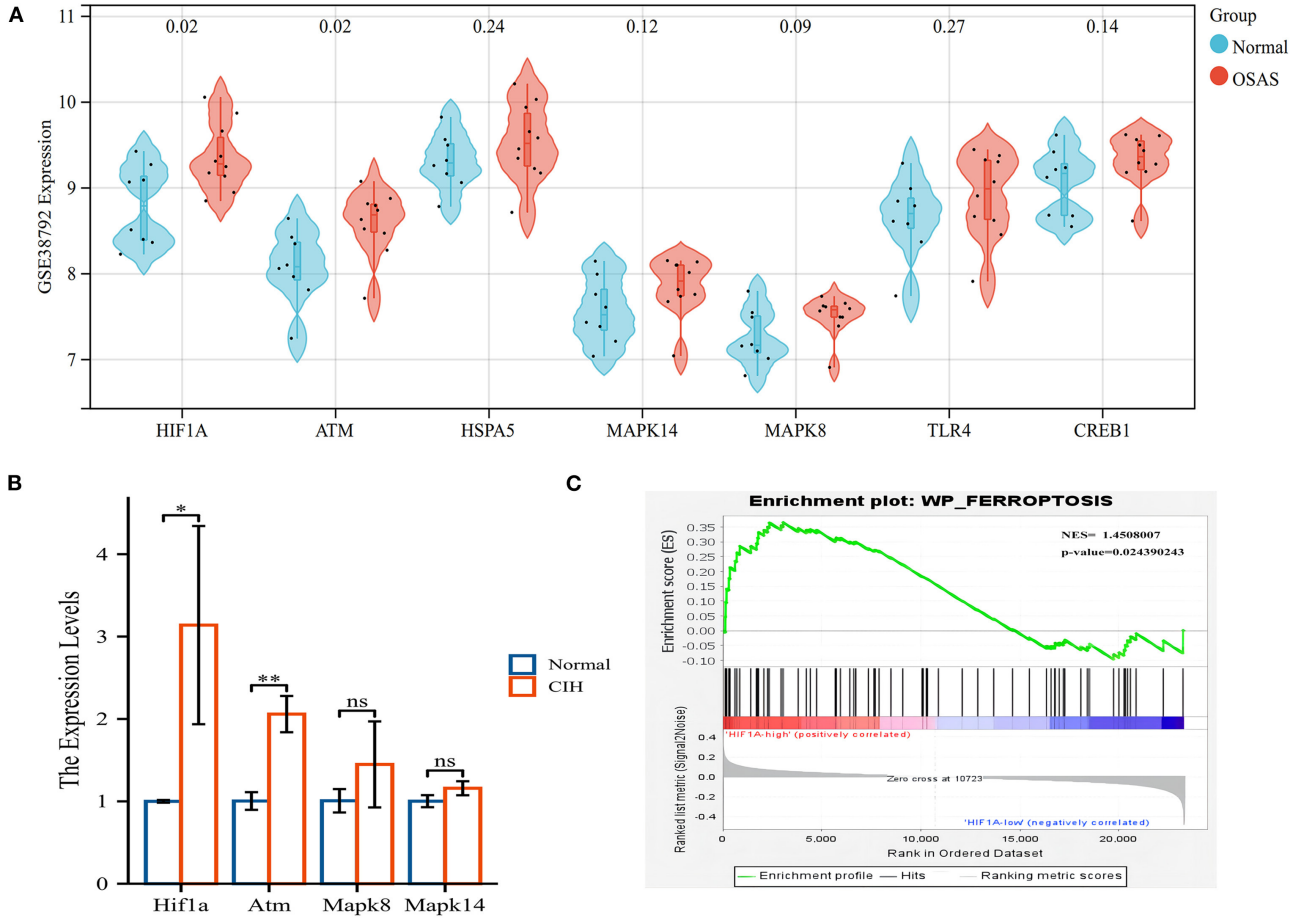
The hub genes (HIF1A and ATM) were validated in another OSAS dataset and the rats qRT-PCR experiment. **(A)** The hub genes were imported into the GSE38792 dataset for validation. **(B)** The mRNA levels of hub genes in the rats of CIH and Normal groups. **(C)** GSEA analysis of HIF1A expression level, NES = 1.4508007, *P* = 0.024. *P < 0.05, **P < 0.01.

We validated the expression of hub genes in another dataset (GSE38792). OSAS had elevated the expression of HIF1A, ATM, HSPA5, MAPK8, MAPK14, TLR4, and CREB1. HIF1A and ATM exhibited statistically significant differences (Figure 6A). HIF1A and Atm mRNA expressions were elevated in rats exposed to CIH, with *P*-values of 0.037 and 0.002, respectively (Figure 6B). Rearrangement of the OSAS phenotype based on HIF1A expression demonstrated that the ferroptosis pathway remained significantly distinct, NES = 1.4508007, *P* < 0.05 (Figure 6C). PO2 (CIH group): 51.86 ± 2.97 mmHg, PO2 (Normal group): 69.87 ± 2.45 mmHg. There is a statistical difference between the two groups (*P* < 0.05).

**Figure 7:**
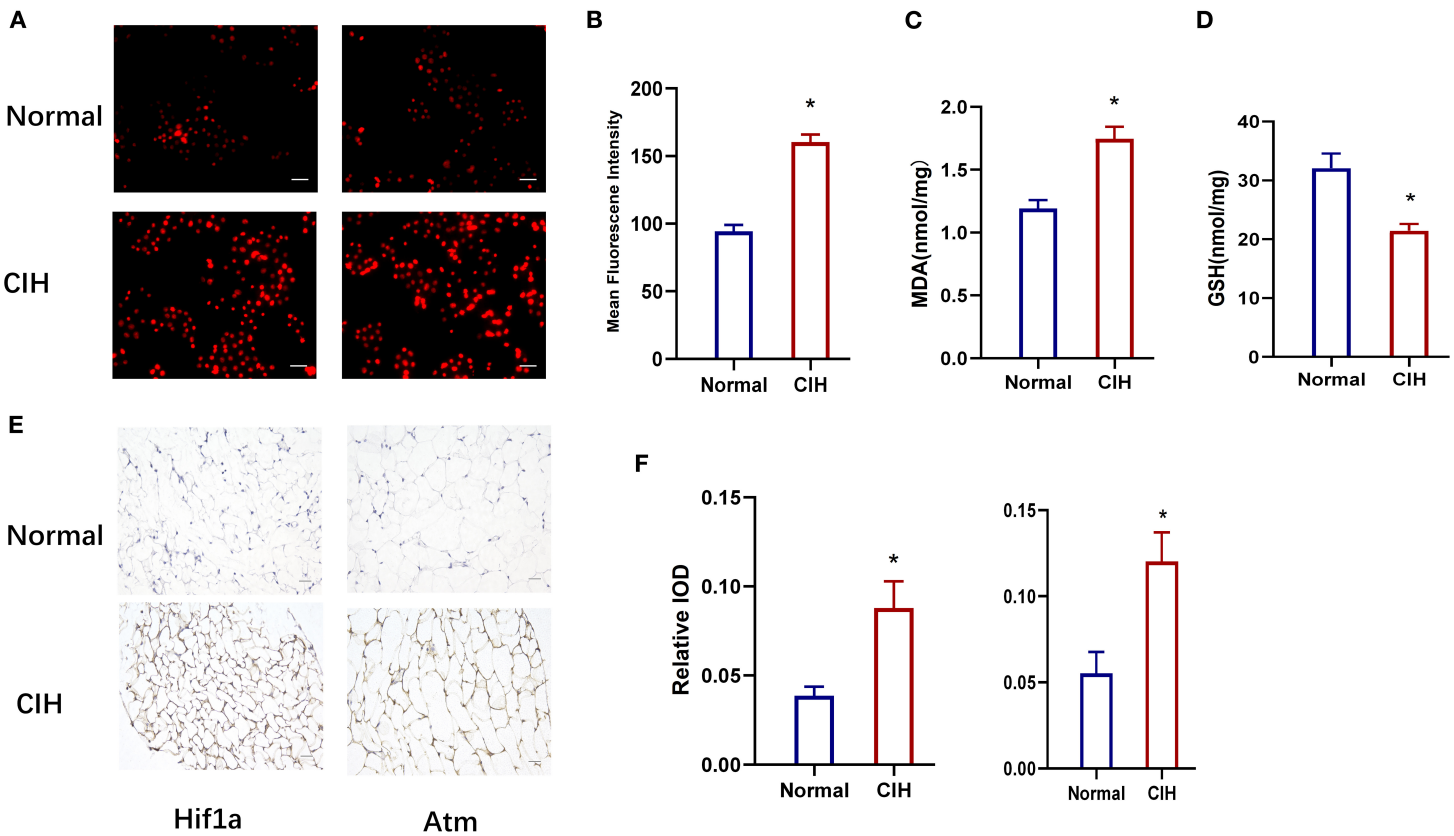
Establishment of chronic intermittent hypoxia model (CIH) in rats, validated by ROS, MDA, GSH, and IHC. **(A, B)** The expression of ROS in rat lung tissue in different groups. Original magnification is × 400, Scare bar, 25 μm. **(C, D)** The expression of MDA and GSH in different groups. **(E, F)** HIF1A and ATM protein expression variations in different groups were detected by immunohistochemistry. Magnification × 200, Scare bar, 100 μm. *P < 0.05.

Figure 7 depicts the values of the oxidative stress markers (ROS, MDA, and GSH) and the hub genes (HIF1A, Atm). CIH exposure increased the expression of ROS in rat lung tissue compared to the control group (Figures 7A, B). MDA was greatly increased in the CIH group compared to the control group, although GSH was dramatically decreased, and the *P-*values were all significant (*P* < 0.01) (Figures 7C, D). The expression of HIF1A and Atm proteins in inguinal fat was examined using immunohistochemical techniques in CIH-exposed rats and was significantly elevated in the CIH group with a significant *P*-value of 0.0049 (Figures 7 E, F). Both Figures 6, 7 demonstrate that HIF1A and ATM play a significant role in the process of OSAS-induced ferroptosis, which is intimately connected to the elevation in ROS levels and the decline in GSH levels.

### ROC diagnostic curve and hub genes relationships

**Figure 8:**
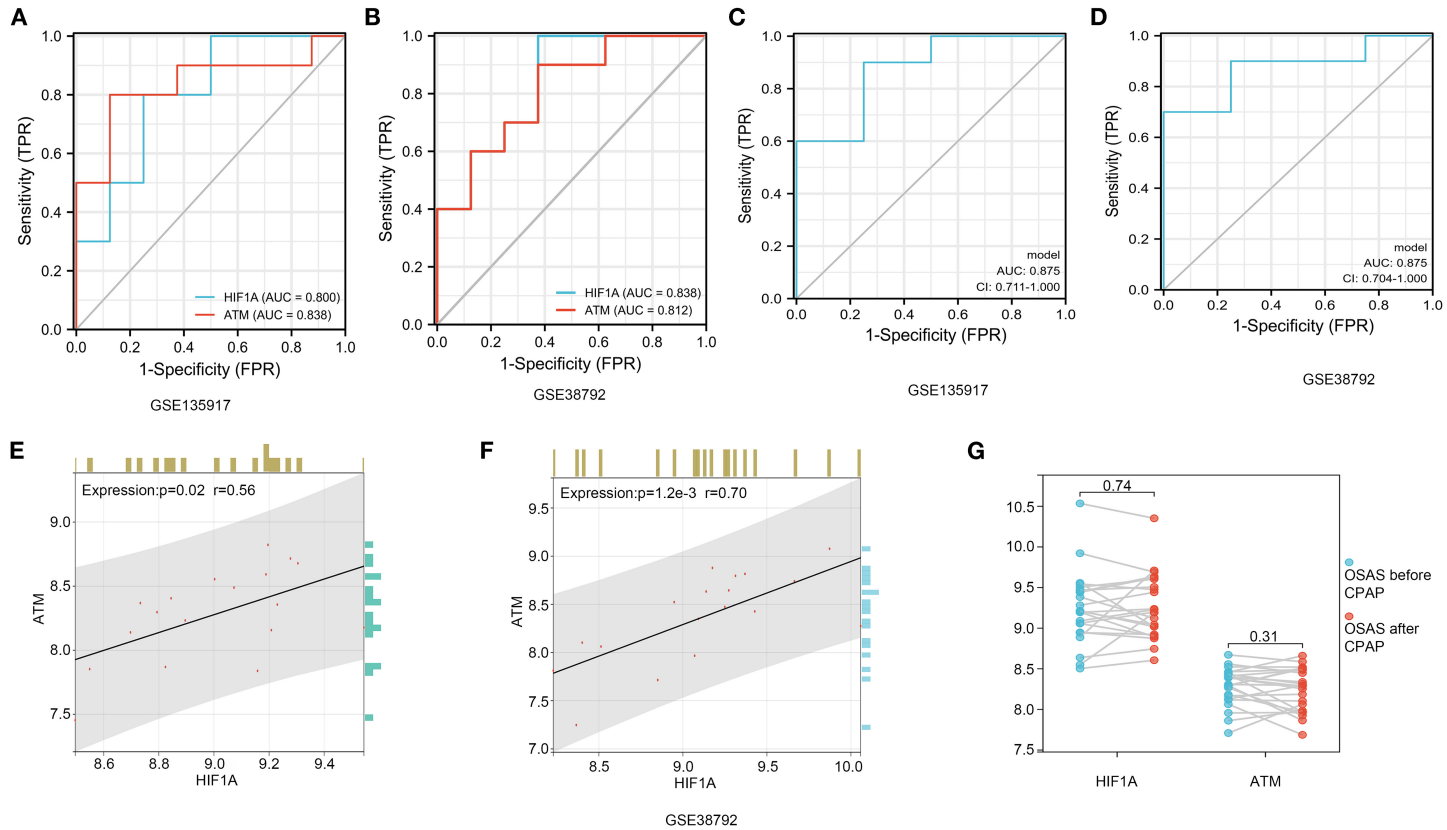
HIF1A and ATM diagnosis curves, expression correlation analysis, and non-invasive ventilator pairing therapy modification. **(A, B)** In GSE135917 and GSE38792 datasets, HIF1A, and ATM expression were examined for ROC. **(C, D)** HIF1A and ATM combined models in GSE135917 and GSE38792 datasets. **(E, F)** The person correlation analysis of HIF1A and ATM. **(G)** Differential expression of HIF1A and ATM before and after non-invasive ventilator (CPAP) treatment in GSE135917.

We performed a diagnostic curve analysis to assess the significance of hub genes (HIF1A and ATM) in the diagnosis of OSAS. The respective areas of HIF1A under the curves (AUC) were 0.838 and 0.80 in both datasets, corresponding to an AUC of 0.838, 0.812 for ATM, respectively (Figures 8A, B). The AUC of the combined HIF1A and ATM model for the diagnosis of OSAS was 0.875 in both datasets, suggesting that the model is beneficial for the identification of OSAS (Figures 8C, D). To assess the interaction analysis between hub genes, Pearson correlation analysis was performed. HIFA and ATM were found to be strongly correlated in both datasets, with correlation coefficients (*r*) of 0.56, 0.70 and *P*-values of 0.02, 1.2e-3, respectively (Figures 8E, F).

It was found that there was no significant change in HIF1A and ATM in OSAS patients treated with ventilators, with *P-*values of 0.74, and 0.31, respectively (Figure 8G). There was no significant change in HIF1A and ATM after short-term non-invasive ventilator (CPAP) therapy, and we investigated pharmacological treatment of OSAS in the following phase.

### Drugs from the drugbank

DrugBank includes the most comprehensive information on medications and their targets, so it provides us with the most important drug targets. Eight drugs targeting these two hub genes were identified from the Drugbank (Table 2). Among these drugs, the modulators are mainly 2-methoxy estradiol, carvedilol, ENMD-1198, and FG-2216, the inducers are hydralazine, and the inhibitors are PX-478 and vadarestat, The stabilizer has caffeine. The approved pharmaceuticals include carvedilol, hydralazine, and caffeine, which are potential OSAS treatments.

**Table 2:** Drugs obtained from the DrugBank database targeting the hub genes.

**Drugbank ID**	**Name**	**Group**	**Target**	**Actions**
DB02342	2-Methoxyestradiol	Investigational	HIF1A	Modulator
DB01136	Carvedilol	Approved, investigational	HIF1A	Modulator
DB05959	ENMD-1198	Investigational	HIF1A	Modulator
DB08687	FG-2216	Investigational	HIF1A	Modulator
DB01275	Hydralazine	Approved	HIF1A	Inducer
DB06082	PX-478	Investigational	HIF1A	Inhibitor
DB12255	Vadadustat	Investigational	HIF1A	Stabilization
DB00201	Caffeine	Approved	ATM	Inhibitor

## Discussion

Chronic intermittent hypoxia is the main pathophysiological mechanism of OSAS. CIH can lead to an increase in ROS, which may result in cellular ferroptosis (20). Ferroptosis was important in CIH-induced liver and cardiac injury (39, 40), however, there are limited investigations on CIH for lung, adipose tissue, and tumor. We analyzed OSAS and ferroptosis-related f-DEGs for functional pathways. The HIF1A and ATM are hub genes in f-DEGs utilizing the PPI network and Cytoscape software.

The KEGG pathway showed that ferroptosis is closely related to autophagy, which is essential for driving cells to undergo ferroptosis. Regulatory mechanisms and signaling pathways for autophagy-dependent ferroptosis may enhance the study of chemo modulators of ferroptosis and may be developed for therapeutic interventions in human diseases (41). Ferroptosis is connected to lysosomes and autophagosomes, according to CC enrichment analysis. The molecular function of ferroptosis may involve DNA transport and activation of the mitogen-activated protein kinase pathway. An anesthetic drug (lidocaine) attenuates pulmonary epithelial cell ferroptosis in a hypoxia/reoxygenation-induced model by modulating the p38 MAPK pathway, suggesting that it is closely related to ferroptosis (42). This is consistent with the enrichment results of f-DEGs in the Disease Ontology database, indicating that CIH may affect the progress of cancers, particularly THCA.

The THCA data was gathered from the TCGA database, the hub genes (HIF1A, ATM) in f-DEGs were investigated, and HIF1A was overexpressed in THCA. There is a lack of research on the relationship between OSAS and thyroid cancer at the present, however, HIF1A is closely associated with both diseases. According to our findings, OSAS can cause CIH, which increases ROS, promotes ferroptosis, and contributes to the onset and progression of THCA. Hypoxia causes cervical lymph node metastases and thyroid cancer recurrence, however, its mechanism is unknown (43). FGF11 interacted with HIF1A to increase thyroid cancer growth and metastasis (44). HIF1A expression correlated positively with Medullary thyroid carcinoma prognosis (MTC) (45). Most likely, the presence of fewer T cells in OSAS patients is due to higher HIF1A levels (46). The analysis of THCA immune cell infiltration revealed a more significant reduction of CD8 T cells (47). OSAS is regarded as a low-grade systemic inflammation triggered by CIH, which can increase the inflammatory factors NF-kB and HIF1A (48). In our research, active CD4 T cells were increased in OSAS, although activated B cells were lower, indicating that OSAS may be associated with immune dysregulation. CIH and chronic inflammation may inhibit the activation of immune cells, especially lymphocytes and monocytes. We believe that the activation of immune cells helps OSAS patients to fight chronic inflammation. However, this may affect their metabolism, such as fat metabolism, and could exacerbate their disease progression. Additionally, it was discovered that an overexpression of HIF1A was associated with decreased immunological activity as well as a decreased survival rate in malignancies (49). Consequently, our findings may demonstrate that HIF1A is probable to cause the progression of OSAS and THCA diseases *via* the ferroptosis mechanism and immunological microenvironment modifies.

The validation in the GSE38792 dataset revealed that the expression of HIF1A and ATM was also elevated. Subsequent immunohistochemistry assays confirmed that HIF1A and Atm proteins were also raised in the adipose tissue of rats. CIH causes a rise in cellular HIF1A, which results in increased NADPH oxidase 4 (NOX4) activity, which can produce more ROS (50). NOX4 promotes ferroptosis-dependent cytotoxicity *via* increasing oxidative stress-induced lipid peroxidation (51). Ataxia-telangiectasia mutated (ATM) is a protein kinase that is necessary for cellular inflammatory toxicity along with oxidative stress-induced cell death (52). Chen et al. identified ATM as the primary ferroptosis kinase using siRNA knockdown (53). Previous research has demonstrated that repeated hypoxic reoxygenation of CIH results in an excessive generation of ROS (54). Our investigation revealed that CIH can not only increase ROS and MDA but also decrease GSH. ROS can cause ferroptosis by activating autophagy and increasing intracellular iron levels through increasing ferritin and transferrin receptors (19).

**Figure 9:**
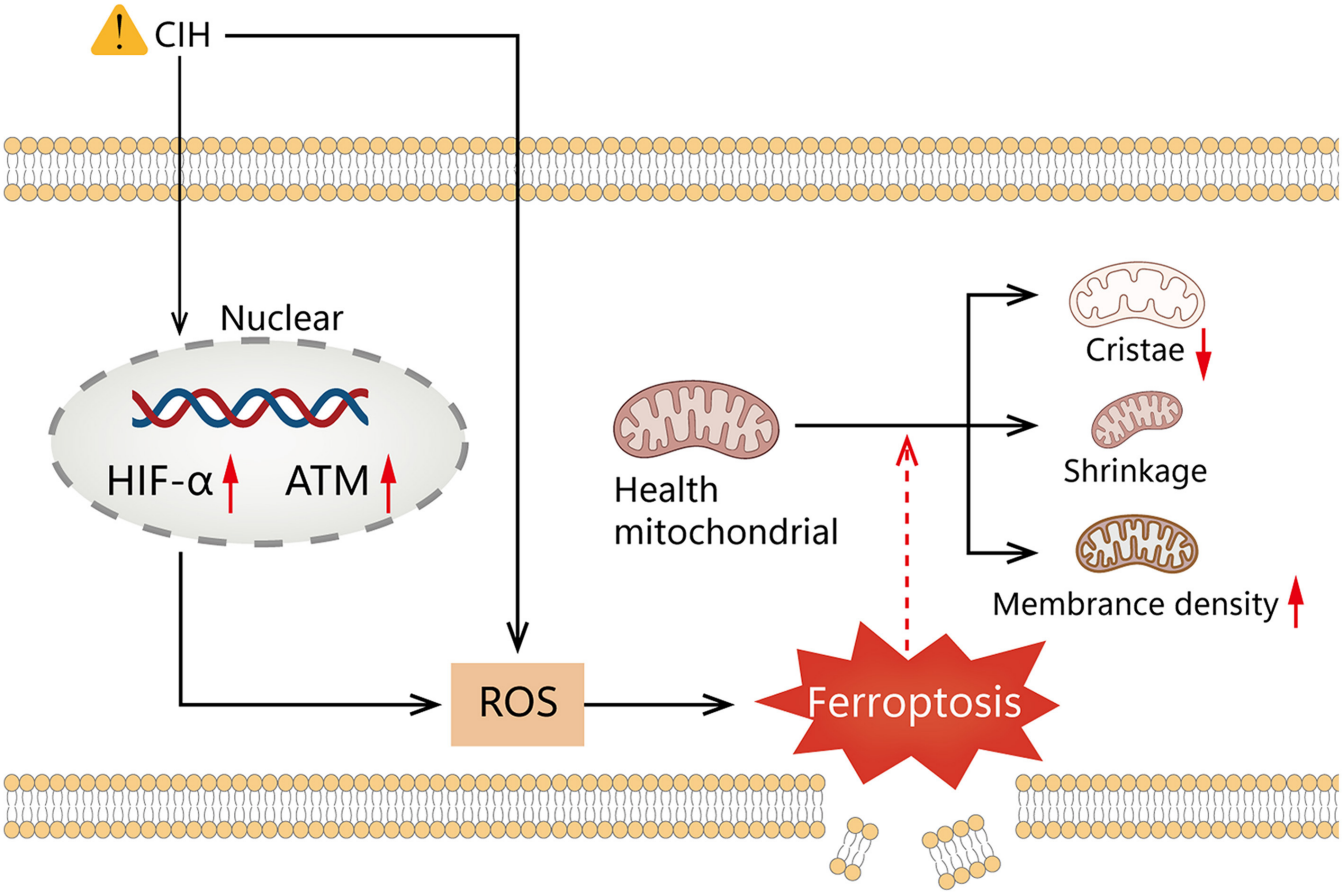
Graphical summary illustrating elevated HIF1A and ATM expression, mediates ROS elevation by chronic intermittent hypoxia promotes ferroptosis.

Our results reveal that HIF1A is significantly positively correlated to ATM in the course of OSAS. Hypoxia can inhibit mTORC signaling, leading to ATM-dependent HIF1A phosphorylation at serine 696 and mediating the downregulation of mTORC1 signaling (55). The ROC curve indicates that the model is advantageous for identifying OSAS. Compared to polysomnography, however, this technique is invasive. Polysomnography is a complicated procedure that is easily influenced by the patient's mental state. The combination of the two may thus aid in the identification of OSAS. It is still debatable whether a ventilator or surgery should be used to treat OSAS (56). Figure 8G shows that 2 weeks of ventilator treatment did not reduce the expression of HIF1A and ATM, implying that CPAP treatment is a long-term procedure. In the DrugBank database, we anticipate discovering drugs that could be utilized to treat OSAS. Hydralazine can reduce the protein levels of HIF1A and its downstream target genes to increase cellular antioxidant capacity (57). CIH can cause raised HIFA and ATM, which can generate high ROS, that can accelerate ferroptosis, resulting in diminished or non-existent mitochondrial cristae, ruptured and contracted outer mitochondrial membranes, and darkened mitochondria (Figure 9).

Our research also has some limitations. Our raw data comes from online databases. The genes that cause ferroptosis are still inadequately known. *In vitro* research aids in elucidating the molecular process underlying ferroptosis.

## Conclusion

Our research discovered that HIF1A and ATM are crucial genes in the process of CIH that leads to ferroptosis and that changes in the immunological microenvironment promote the progression of tumor disorders such as THCA. At present, the main method for diagnosing OSAS is polysomnography, but there is a lack of specific markers. The hub genes (HIF1A and ATM) can serve as biomarkers and therapeutic targets for OSAS. Therefore, this study may provide new insights into the role of ferroptosis in the pathogenesis of OSAS.

## Data availability statement

The original contributions presented in the study are included in the article/Supplementary material, further inquiries can be directed to the corresponding author.

## Ethics statement

This research was carried out in accordance with the Regulations of Experimental Animal Administration issued by the State Committee of Science and Technology of the People's Republic of China, with the approval of the Ethics Committee in Renmin Hospital of Wuhan University(IACUC Issue No: 20220501A).

## Author contributions

All authors listed have made a substantial, direct, and intellectual contribution to the work and approved it for publication.

## Supplementary material

Supplementary Figure S1 GSE135917 data preprocessing. **(A)** RNA degradation Degradation in the 5' → 3' direction in the mRNA molecular degradation pathway. **(B)** 2-dimensional of PLS-DA. **(C)** 3-dimensional of PLS-DA. **(D)** PLS-DA permutation test 200 permutations 4 components.

Supplementary Figure S2 Immune cell analysis of OSAS disease. **(A)** GSVA immune cell analysis calculation of various immune cell scores for each sample by gsva method. **(B)** Various immune cell correlation heat maps in OSAS samples and Normal controls. **(C)** The hub genes associated with immune cells in OSAS samples and Normal controls. **(D)** HIF1A is associated with immune cells.

Supplementary Table S1 KEGG pathway analysis.
