# Supplementary figures and images for: Identification and validation of ferroptosis-related hub genes in obstructive sleep apnea syndrome
Source: Front Neurol. 2023 Mar 2;14:1130378. doi: 10.3389/fneur.2023.1130378 (PMC10018165; doi:10.3389/fneur.2023.1130378)

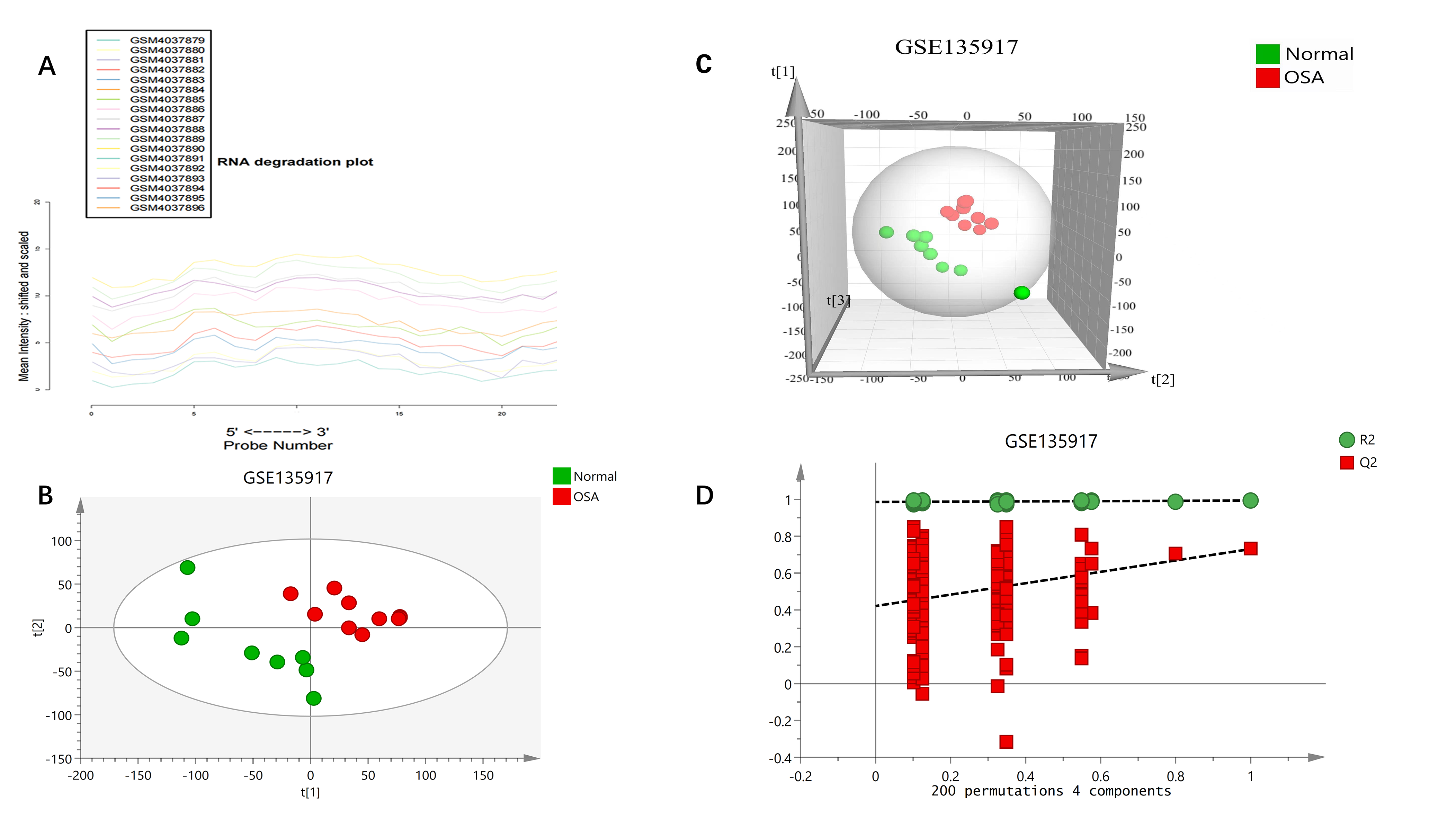

Supplement: Supplementary Figure S1 — GSE135917 data preprocessing. (A) RNA degradation Degradation in the 5' → 3' direction in the mRNA molecular degradation pathway. (B) 2-dimensional of PLS-DA. (C) 3-dimensional of PLS-DA. (D) PLS-DA permutation test 200 permutations 4 components. [file Image_1.TIF]

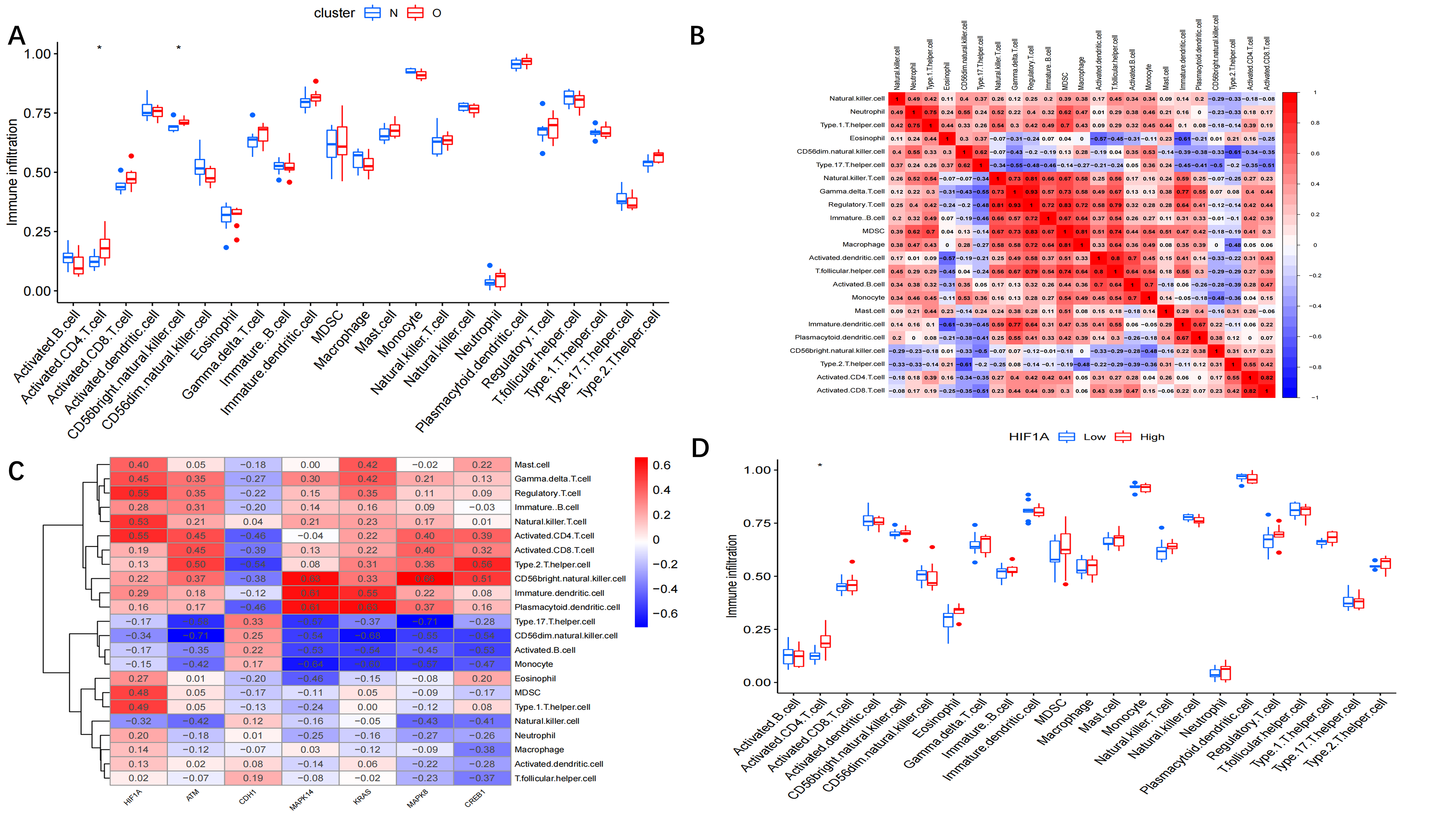

Supplement: Supplementary Figure S2 — Immune cell analysis of OSAS disease. (A) GSVA immune cell analysis calculation of various immune cell scores for each sample by gsva method. (B) Various immune cell correlation heat maps in OSAS samples and Normal controls. (C) The hub genes associated with immune cells in OSAS samples and Normal controls. (D) HIF1A is associated with immune cells. [file Image_2.TIF]
