# Supplementary material for: Identification and validation of ferroptosis-related hub genes in obstructive sleep apnea syndrome
Source: Front Neurol. 2023 Mar 2;14:1130378. doi: 10.3389/fneur.2023.1130378 (PMC10018165; doi:10.3389/fneur.2023.1130378)
Supplement: Supplementary Table S1 — KEGG pathway analysis. [file Table_1.docx]

**Table S1** KEGG KEGG pathway analysis

| **ID** | **Description** | **GeneRatio** | **BgRatio** | **p-value** | **p.adjust** | **qvalue** |
| --- | --- | --- | --- | --- | --- | --- |
| hsa05161 | Hepatitis B | 13/53 | 162/8076 | 2.21e-11 | 4.41e-09 | 2.02e-09 |
| hsa04140 | Autophagy -animal | 10/53 | 137/8076 | 1.49e-08 | 1.49e-06 | 6.81e-07 |
| hsa05167 | Kaposi infection | 11/53 | 193/8076 | 3.47e-08 | 2.31e-06 | 1.06e-06 |
| hsa04621 | NOD-like receptor | 10/53 | 181/8076 | 2.11e-07 | 9.93e-06 | 4.55e-06 |
| hsa04137 | Mitophagy - animal | 7/53 | 68/8076 | 2.48e-07 | 9.93e-06 | 4.55e-06 |
| hsa04622 | RIG-I-like receptor | 7/53 | 70/8076 | 3.04e-07 | 1.01e-05 | 4.64e-06 |
| hsa05170 | HIV-1 infection | 10/53 | 212/8076 | 9.14e-07 | 2.61e-05 | 1.20e-05 |
| hsa01522 | Endocrine resistance | 7/53 | 98/8076 | 3.05e-06 | 7.62e-05 | 3.49e-05 |
